# Silencing of ATP2B1‐AS1 contributes to protection against myocardial infarction in mouse via blocking NFKBIA‐mediated NF‐κB signalling pathway

**DOI:** 10.1111/jcmm.15105

**Published:** 2020-03-10

**Authors:** Kai‐You Song, Xian‐Zhao Zhang, Feng Li, Qing‐Rong Ji

**Affiliations:** ^1^ Department of Cardiology Linyi People's Hospital Linyi China; ^2^ Clinical Laboratory The Third People's Hospital of Linyi Linyi China

**Keywords:** mouse ATPase plasma membrane Ca^2+^ transporting 1 antisense RNA 1, myocardial infarction, NF‐kappa‐B inhibitor alpha, nuclear factor‐kappa‐B signalling pathway

## Abstract

Myocardial infarction (MI) is an acute coronary syndrome that refers to tissue infarction of the myocardium. This study aimed to investigate the effect of long intergenic non‐protein‐coding RNA (lincRNA) ATPase plasma membrane Ca^2+^ transporting 1 antisense RNA 1 (ATP2B1‐AS1) against MI by targeting nuclear factor‐kappa‐B inhibitor alpha (NFKBIA) and mediating the nuclear factor‐kappa‐B (NF‐κB) signalling pathway. An MI mouse model was established and idenepsied by cardiac function evaluation. It was determined that ATP2B1‐AS1 was highly expressed, while NFKBIA was poorly expressed and NF‐κB signalling pathway was activated in MI mice. Cardiomyocytes were extracted from mice and introduced with a series of mouse ATP2B1‐AS1 vector, NFKBIA vector, siRNA‐mouse ATP2B1‐AS1 and siRNA‐NFKBIA. The expression of NF‐κBp50, NF‐κBp65 and IKKβ was determined to idenepsy whether ATP2B1‐AS1 and NFKBIA affect the NF‐κB signalling pathway, the results of which suggested that ATP2B1‐AS1 down‐regulated the expression of NFKBIA and activated the NF‐κB signalling pathway in MI mice. Based on the data from assessment of cell viability, cell cycle, apoptosis and levels of inflammatory cytokines, either silencing of mouse ATP2B1‐AS1 or overexpression of NFKBIA was suggested to result in reduced cardiomyocyte apoptosis and expression of inflammatory cytokines, as well as enhanced cardiomyocyte viability. Our study provided evidence that mouse ATP2B1‐AS1 silencing may have the potency to protect against MI in mice through inhibiting cardiomyocyte apoptosis and inflammation, highlighting a great promise as a novel therapeutic target for MI.

## 1 INTRODUCTION

Myocardial infarction (MI) is a major public health concern that is commonly known as a heart attack characterized by a decline or complete stop of blood flow to the heart.^1^ Since 1998, age‐standardized AMI incidence decreased from 323 to 210 per 100 000 in 2007 in female and from 620 to 380 per 100 000 in 2007 in male, but socio‐economic inequalities still exist in AMI incidence and have not narrowed.^2^ In America, more than one million patients are hospitalized due to MI annually, and the rates of readmission are approximately 20% within 30 days after discharge.^3^ Moreover, the incidence of MI is increasing in China, and 23 million patients are estimated to experience MI by the end of 2030.^4^ The risk factors of MI include hypertension, hypercholesterolaemia, diabetes, smoking habit, obesity, a sedentary lifestyle, excessive alcohol intake and unhealthy diet.^5^, ^6^ Earlier recognition or diagnosis of MI symptoms and prompt care‐seeking actions are of great significance for the selection of the most appropriate treatments.^7^ Although great improvements have been achieved in the prevention and therapies of MI, the difficulties in the prevention of MI are still steadily increasing.^8^ Thus, further efforts are needed to develop more promising and effective therapeutic strategies for MI.

Long non‐coding RNAs (lncRNAs) constitute a group of non‐coding RNAs implicated in diverse biological processes through regulation of gene expression and have recently been reported to be novel predictors in the setting of AMI.^9^ ATPase plasma membrane Ca^2+^ transporting 1 antisense RNA 1 (ATP2B1‐AS1), also named long intergenic non‐protein‐coding RNA 936 (LINC00936), has been found to be an important regulator in chronic renal failure‐induced renal interstitial fibrosis and oxidative stress.^10^ However, the relationship between this lncRNA and MI remains to be defined.

Nuclear factor‐kappa‐B inhibitor alpha (NFKBIA), also known as IκBα, has the great potency to suppress nuclear factor‐kappa B (NF‐κB).^11^ NF‐κB is a set of transcription factors, including RelB, c‐Rel, NF‐κB1 (p50), NF‐κB2 (p52) and RelA (p65), that critically function as regulators in cell growth, cell apoptosis and immune inflammatory responses.^12^ NF‐κB has been shown to regulate approximately 200 target genes, and most of them are involved in inflammation.^13^ In addition, NF‐κB still exerts impacts on oxidative stress, genotoxic stress and the self‐regulation of homeostatic functions.^14^ Activated NF‐κB signalling pathway can be observed in myocardial tissues from AMI mouse model, while impaired NF‐κB signalling pathway could impede the cardiac rupture and ventricular remodelling.^15^ However, investigations on the roles of lncRNAs and NF‐κB and their interaction in MI are still limited. In the present study, we investigated the roles of mouse ATP2B1‐AS1 in MI through the regulation of the NFKBIA expression and NF‐κB signalling pathway in mice.

## 2 MATERIALS AND METHODS

### 2.1 Ethics statement

All experiments were performed in compliance with the Guide for the Care and Use of Laboratory Animals and approved by the Experimental Animal Ethics Committee of Linyi People's Hospital.

### 2.2 In silico analysis

The microarray data and annotated probe files of microarray data sets (http://www.ncbi.nlm.nih.gov/geo/query/acc.cgi?acc=GSE66360, http://www.ncbi.nlm.nih.gov/geo/query/acc.cgi?acc=GSE48060, http://www.ncbi.nlm.nih.gov/geo/query/acc.cgi?acc=GSE65705 and http://www.ncbi.nlm.nih.gov/geo/query/acc.cgi?acc=GSE97320) were downloaded from the Gene Expression Omnibus (GEO) database (http://www.ncbi.nlm.nih.gov/geo/), which were normalized and corrected using the Affy package of the R software.^16^ Then, linear models with empirical Bayes moderation (Limma) combined with *t*‐statistics were performed for the microarray analysis in order to select the differentially expressed long non‐coding RNAs (lncRNAs).^17^ Prediction of the differentially expressed lncRNA expression was performed using Multi‐Experiment Matrix (MEM) database (http://biit.cs.ut.ee/mem/).^18^ Based on the WebGestalt (http://www.webgestalt.org) database, a Kyoto Encyclopedia of Genes and Genomes (KEGG) enrichment analysis of the candidates was proceeded to determine their involvement in major physiological metabolic processes and signalling pathways.^19^ The lncRNA targets were predicted on a website available at http://www.herbbol.org:8001/lrt/index.php.^20^

### 2.3 Sequence analysis

The conserved sequences of homologous genes among different species shared some similarities. The nucleotide sequence of the full‐length LINC00936 gene for human (available at https://www.ncbi.nlm.nih.gov/nuccore/NR_028138.1) was used as the query sequence to search the sequence of mice from genomic database (GRCm38/mm10) in UCSU (http://genome.ucsc.edu/) using basic local alignment search tool (BLAST), and the homologous sequences of LINC00936 gene in human and mouse were compared. The conserved nature of the lncRNA gene between two species was analysed.

### 2.4 Establishment of a mouse model of MI

Forty‐five adult male C57BL/6 mice (aged 12 weeks, weighing 25‐30 g) were provided by the Animal Lab Center of Capital Medical University (Beijing, China). All mice were acclimatized for 1 week in 22‐26°C under a 12/12‐hours light/dark cycle with free access to food and water. Thirty mice were randomly selected for the establishment of the MI model.^21^ The mice were anaesthetized by intraperitoneal administration of 1% pentobarbital sodium (P3761, 50 mg/kg; Sigma‐Aldrich Chemical Company) and placed in the supine position on a heating pad. The mice were intubated and connected to a rodent ventilator (SAR‐830/P; CWE Inc) at a respiratory rate of 30 breaths/min and tidal volume of 7 mL/kg. Following a left thoracotomy at the fourth intercostal space, the left thoracic cavity was exposed, and ligation of the left anterior descending (LAD) coronary artery was performed 2‐3 mm around the lower edge of the left atrial appendage using an 8‐0 atraumatic suture. The immediate colour change in the heart surface, which turned dark red, and the ST‐segment elevation in leads II were signs of a successful coronary ligation. The chest was then closed and sutured. Mice received an intramuscular injection of penicillin (130 437; BIOBW Biological Technology Co., Ltd.) at a dosage of 10 000 U/d for three consecutive days. During the recovery period, the mice were allowed to breath spontaneously without tracheal intubation and placed on a heating pad maintained at 39°C. The remaining fifteen mice were sham‐operated with the same procedures without LAD ligation. The infarct area was pale in the visual inspection during the surgery, and electrocardiogram (ECG) was recorded before and after the surgery.

### 2.5 Ultrasonic cardiogram (UCG) test

After anaesthesia with pentobarbital sodium, the mice were fixed in the supine position before surgery and 2 weeks after surgery, respectively. An ultrasonic instrument (ACUSON, Siemens) was used for the cardiac function assessment at 13 mHz. M‐mode image of a longitudinal section of the left ventricular papillary muscles in the anterior chest area was captured in the short‐axis using ultrasonic coupling gel. The left ventricular end‐diastolic diameter (LVEDD) and left ventricular end‐systolic diameter (LVESD) in three cardiac cycles were measured separately and averaged. Left ventricular fractional shortening (LVFS) = (LVEDD − LVESD)/LVEDD × 100%.^22^

### 2.6 Haematoxylin‐eosin (HE) staining

HE staining was performed to observe the MI‐induced histological changes.^23^ Two weeks after surgery, the mice from the MI and sham groups were anaesthetized with pentobarbital sodium. Then, heart obtained by thoracotomy was rinsed with phosphate buffered saline (PBS), fixed with 10% formalin for 12 hours and dissected along the ligature to excise myocardial tissues. The paraffin‐embedded tissues were sliced into 4‐μm sections. After being heated at 60°C, the sections were dewaxed with xylene for 20 minutes, dehydrated using gradient ethanol and soaked in distilled water for 5 minutes. Afterwards, the sections were stained with haematoxylin (H8070; Solarbio) for 4 minutes and counterstained with eosin (E8090; Solarbio) for 3 minutes. Finally, the sections were observed under an optical microscope (DSX100, Olympus) and photographed.

### 2.7 2, 3, 5‐triphenyltetrazolium chloride (TTC) staining

The infarct size (IS) was measured by conducting TTC staining.^23^, ^24^ The heart was isolated and washed with PBS, and the atrium and right ventricle were removed. The left ventricle was weighed and stored at −20°C for 20 minutes. Subsequently, the left ventricle was sliced into 2‐mm sections and stained with 1% TTC solution (MHB0268a; Yuanye Biotechnology Co., Ltd.) at 37°C for 10 minutes. Then, the sections were dried, weighed and fixed with 4% paraformaldehyde (P1110, Solarbio) for 30 minutes. The IS was analysed using Image‐Pro Plus software (IPP 7.0, Media Cybernetics) and calculated as follows: IS(%)=theweightoftheinfractedmycardium(IMW)/theweightoftheleftventricular(LVW)×100%..

### 2.8 Terminal deoxynucleotidyl transferase–mediated dUTP nick‐end labelling (TUNEL) staining

In compliance with the specifications of TUNEL Kit (C1086; Beyotime Biotechnology Co.), cell apoptosis was observed. Paraffin sections were dewaxed, rehydrated and immersed in 3% H_2_O_2_ for 10 minutes at ambient temperature. The sections were hydrolysed with 50 μL of 20 μg/mL proteinase K (P6556; Sigma‐Aldrich) for 20 minutes. The citrate was added for 30 minutes of antigen retrieval. Subsequently, the sections reacted with 50 μL deoxynucleotide transferase (TdT) in a wet box in the dark at 37°C for 1 hour. The TdT‐free TUNEL reaction solution was used as a negative control (NC). Then, the sections were reacted with 50 μL peroxidase‐labelled anti‐digoxigenin in a wet box at 37°C in the dark for 30 minutes. The sections were stained with 4',6‐diamidine‐2‐phenylindole (DAPI) solution (C1002; Beyotime Biotechnology Co.) for 10 minutes. Each step was followed by PBS washing. Finally, the sections were sealed using neutral balsam and observed under a fluorescence microscope (BX53; Olympus). In ten randomly selected visual fields, positive cells and the total cells were counted. The ratio (positive cells/total cells) represented the apoptosis index (AI).

### 2.9 Immunohistochemical staining

After dewaxing and hydration of paraffin‐embedded sections, the antigen retrieval was conducted for 10 minutes using 0.01 M sodium citrate buffer. The sections were subsequently immersed in 0.3% H_2_O_2_‐methanol solution for 20 minutes. After PBS rinsing, the sections were blocked with 10% goat serum (36119ES03; Yeasen Biotechnology Co., Ltd.) for 10 minutes. The sections were then incubated with a rabbit antimouse antibody to NFKBIA (1:100; ab32518; Abcam Inc) or PBS as the NC, at 4°C overnight. The sections were incubated with horseradish peroxidase (HRP)‐labelled goat anti‐rabbit to IgG (1:1000; ab6721; Abcam) for 30 minutes. Development with 3, 3′‐diaminobenzidine (DAB; P0203; Beyotime) was performed for 5 minutes. After counterstaining with haematoxylin for 3 minutes, the sealed sections were observed under a fluorescence microscope. Five randomly selected fields of each tissue were observed to calculate the mean optical density (OD) using Image‐Pro 7.0 software.

### 2.10 Reverse transcription quantitative polymerase chain reaction (RT‐qPCR)

The total RNA was extracted from the myocardial tissues and cells using a TRIzol Kit (15596‐026; Invitrogen). The concentration and purity of extracted RNA were detected using a nucleic acid protein determinator (BioPhotometer D30, Eppendorf). The RNAs were reversely transcribed into cDNA by a Reverse Transcription Kit (K1621; Fermentas). The primers of the mouse ATP2B1‐AS1, NFKBIA, NF‐κBp50, NF‐κBp65, IKKβ and β‐actin (Table 1) were designed and synthesized by Shanghai Genechem Co., Ltd. The mRNA levels of the genes were determined by a fluorescent quantitative PCR kit (Takara). The qPCR was performed using an ABI 7500 PCR instrument (ABI). Using β‐actin as an internal reference, 2^‐ΔΔCT^ was used to calculate the relative target gene expression as follows: $\Delta \Delta \text{CT}=\left(\Delta {\text{Ct}}_{\text{target}\;\text{gene}\;\text{in}\;\text{experimental}\;\text{group}}-\Delta Ct_{\text{housekeeping}\;\text{gene}\;\text{in}\;\text{experimental}\;\text{group}}\right)-\left(\Delta Ct_{\text{target}\;\text{gene}\;\text{in}\;\text{control}\;\text{group}}-\Delta Ct_{\text{housekeeping}\;\text{gene}\;\text{in}\;\text{control}\;\text{group}}\right)$.

**Table 1:** Primer sequences for RT‐qPCR

Gene	Primer sequence (5′‐3′)
mouse ATP2B1‐AS1	Forward: GCTCTGACGTCTGTGTTTCCA
	Reverse: AAGTGAAGGGCGTCCCACT
NFKBIA	Forward: AGCACAAAGAGAGTGTCGC
	Reverse: CAGCGTTCATGGTTATGG
NF‐κBp50	Forward: GCTTACGGTGGGATTGCATT
	Reverse: GGCACAATCTCTAGGCTCGTTT
NF‐κBp65	Forward: TCACCAAAGACCCACCTCACCG
	Reverse: GGACCGCATTCAAGTCATAGTCCC
IKKβ	Forward: GTGGACATCGTTGTTAGT
	Reverse: AAGACACTGTTAAGATTATTGG
β‐actin	Forward: CGTGGGCCGCCCTAGGCACCA
	Reverse: GGGGGGACTTGGGATTCCGGTT

Abbreviations: ATP2B1‐AS1, ATPase plasma membrane Ca^2+^ transporting 1; NFKBIA, NF‐kappa‐B inhibitor alpha; RT‐qPCR, reverse transcription quantitative polymerase chain reaction.

### 2.11 Western blot analysis

An amount of 50 mg myocardial tissue samples were extracted and lysed in protein lysis buffer (R0010; Solarbio), and centrifuged at 3000 g. After an ice bath for 30 minutes, the supernatant obtained by centrifugation at 5000 g at 4°C for 15 minutes were harvested for further use. The protein concentration was determined using a bicinchoninic acid protein assay kit (23225; Pierce) and adjusted to 1 μg/μL. The proteins were separated with 10% sodium dodecyl sulphate‐polyacrylamide gel electrophoresis (P1200; Solarbio). Subsequently, the proteins were transferred onto the polyvinylidene fluoride membranes (HVLP04700; Millipore). The membranes were blocked with 5% skim milk powder at ambient temperature for 2 hours. The membranes were incubated with primary antibodies, including rabbit antibodies to NFKBIA (1:1000, ab32518), NF‐κBp50 (1:1000, ab32360), NF‐κBp65 (1:50 000, ab32536), IKKβ (1:1000, ab32135) and β‐actin (1:1000, ab8227) at 4°C overnight. HRP‐labelled goat anti‐rabbit IgG (1:2000, ab6721) served as secondary antibody for a 2‐hours incubation. Each step was followed with Tris‐buffered saline with Tween 20 (TBST) washing. All antibodies were purchased from Abcam Inc. After the DAB staining, the proteins were photographed using a gel imager (Gel Doc XR, Bio‐Rad). β‐actin was utilized as internal control. The experiment procedures were also suitable for the measurement of cellular proteins.

### 2.12 Isolation of adult mouse cardiomyocytes

The adult mouse cardiomyocytes were isolated using the method outlined in a previous study.^25^ In details, the mice were intraperitoneally injected with heparin and killed by cervical dislocation, followed by heart extraction. The heart was rinsed with Joklik's modified minimal essential medium, and tubes were inserted into the aorta. After perfusion at 37°C, the aorta was rinsed for 5 minutes, followed by circulatory perfusion with 40 mL digestive enzyme solution. After centrifugation at 1000 g for 10 minutes, the precipitation (cells) was resuspended with M199 culture medium containing 10% foetal bovine serum (FBS) and cultured in a 5% CO_2_ incubator. The culture medium was renewed every two days. The observation was conducted under an inverted microscope (IX53; Olympus). After fixation with 10% formalin for 30 minutes, the cells were incubated in 0.1% Triton X‐100 (B1276; Westang Biotechnology Co., Ltd.) and 1% FBS for 1 hour. Subsequently, the cells were incubated at 4°C with the rabbit antibody to cTnT (1:400) overnight, followed by incubation with diluted fluorescent secondary antibodies (1:400) at 37°C for 30 minutes. The cells were then stained with DAPI (1:20) at 37°C for 30 minutes. PBS washing was conducted after each step. Finally, the cells were visualized under the fluorescence microscope.

### 2.13 Isolation of neonatal mouse cardiomyocytes

The neonatal mouse cardiomyocytes were isolated as previously described.^26^ In brief, hearts of 1‐day‐old neonatal mice (aged 1 day) were detached, and the myocardial tissues were extracted. The tissues were minced into 1 mm^3^ tissue blocks and detached with 0.1% collagenase I and 0.01% trypsin in a 37°C water bath for 80 minutes. The tissues were detached again with 2 mL digestive enzyme in a 37°C water bath for 30 minutes and cultured with 3 mL M199 medium (31100035; Gibco) containing 10% FBS (16000‐044; Gibco).

### 2.14 Isolation of myocardial fibroblasts

The mouse hearts were extracted, and the ventricle was minced into 1‐mm^3^ tissue blocks. The tissue blocks were placed in Dulbecco's modified eagle's medium (DMEM)/F12 and incubated with 0.25 mg/mL collagenase II at 37°C for 35 minutes. The tissues blocks were gently triturated, and the obtained cell suspension was filtered using cell strainers. The filtered cell suspension was centrifuged at 4°C and 1000 g for 5 minutes, followed by removal of supernatant. The unattached cells were discarded after incubation at 37°C with 50 mL/L CO_2_. Then, the cells were incubated with 10 mL DMEM/F12 containing 100 mL/L FBS, and the medium was renewed every 3 days.

### 2.15 Isolation of endothelial cells

The mice were killed by cervical dislocation, immersed in 75% ethanol for disinfection and fixed on an operation table. The pleuroperitoneal cavity was exposed through thoracolaparotomy, and the aorta was found on the left side of the spine. The thoracic and abdominal aortas were completely extracted and immediately placed in the DMEM, and then detached using 15 mL collagenase (180 ~ 200 U/mL) at 37°C for 45 minutes. The cells filtered through a 70‐μM cell strainer were centrifuged at 4°C for 10 minutes. After 24 hours, the adherent cells were removed. Finally, the cells were seeded into a monolayer at the bottom of the well and sub‐cultured.

### 2.16 Isolation of peripheral blood mononuclear cells (PBMCs)

The cardiac valves of the mice were punctured to collect 0.5 ~ 1 mL blood. Anticoagulant blood was uniformly mixed with the same volume of Royal Park Memorial Institute 1640. The sample was added with lymphocyte separation medium and centrifuged at 600 g for 30 minutes. The monocyte layer was removed. After cell suspension, the cells were seeded in a 24‐well plate at 2 × 10^6^ cells/well and cultured at 37°C with 5% CO_2_ for 2 ~ 4 hours. The non‐adherent cells were discarded. Finally, the purified PBMCs were obtained through the adherent method.^27^

### 2.17 Cardiac troponin T (cTNT) fluorescent staining

Cardiomyocytes were seeded in a 24‐well plate at the density of 1 × 10^5^ cells/well. The cells were incubated at 37°C for 12 hours. The cells were fixed with 10% paraformaldehyde for 30 minutes and incubated with sealing solution containing 0.1% Triton X‐100 (B1276, Westang) and 1% FBS at ambient temperature for 1 hour. Subsequently, the cells were incubated with mouse antibody to cTNT (ab8295, 1:200, Abcam Inc) at 4°C overnight. Then, the cells were incubated with diluted secondary fluorescent antibody (1:400) at 37°C for 30 minutes. The cells were stained with diluted DAPI (1:20) at 37°C for 30 minutes. Finally, the stained cells were visualized under the fluorescence microscope.

### 2.18 Construction of the overexpression and interference plasmids

According to the known transcription sequences in the GenBank database, the full‐length sequences of mouse ATP2B1‐AS1 and NFKBIA, small interfering RNA (siRNA) sequences (siRNA‐mouse ATP2B1‐AS1‐1: CCTCCCAAGTTCAAACAAT, siRNA‐mouse ATP2B1‐AS1‐2: GCCATTACGGCTCAATGCA, siRNA‐mouse ATP2B1‐AS1‐3: GACAATCAGGCATGTGCTT; siRNA‐NFKBIA‐1: GCTGCGATGCATCTTTGGT, siRNA‐NFKBIA‐2: TCTTCCGTCGCTTGAGGAA, siRNA‐NFKBIA‐3: CCATATCGCCTGCAGGAAA) and non‐sense NC sequences (siRNA‐MOUSE ATP2B1‐AS1‐NC: CCAAGCATCCAAATCTTCA; siRNA‐NFKBIA‐NC: ATCGCCAGGGAACATCCTA) were designed using the Ambion website and synthesized by Genechem. The synthesized sequence was cloned into the plasmid vector pcDNA3.1 (VPI0001; Invitrogen Corporation) using cleavage sites of Hind III and XHo I and ligated at 16°C for 1 hour. Then, the ligated products were transfected into the E‐coli DH5α cells (D9052, Takara Biotechnology Ltd.), and the resistant colonies were selected for the PCR determination. In addition, the positive colonies were sequenced. Finally, the extracted plasmids were stored at −20°C for further use.

### 2.19 Cell transfection and grouping

The cardiomyocytes at passage 3 were grouped as follows: blank group (without transfection), NC group (transfected with the NC plasmid), mouse ATP2B1‐AS1 vector group (transfected with the mouse ATP2B1‐AS1 overexpression plasmid), NFKBIA vector group (transfected with the NFKBIA overexpression plasmid), siRNA‐mouse ATP2B1‐AS1 group (transfected with the siRNA‐mouse ATP2B1‐AS1 plasmid), siRNA‐NFKBIA group (transfected with the siRNA‐NFKBIA), mouse ATP2B1‐AS1 vector + NFKBIA vector group (cotransfected with the mouse ATP2B1‐AS1 and NFKBIA overexpression plasmids) and siRNA‐mouse ATP2B1‐AS1 + siRNA‐NFKBIA group (cotransfected with the siRNA‐mouse ATP2B1‐AS1 and siRNA‐NFKBIA plasmids). The trypsinized cells were resuspended with M199 medium containing 10% FBS into 1 × 10^5^ cells/mL and seeded in 6‐well plates. Once the cells reached 70% confluence, the cells were cultured in serum‐free M199 medium. An oxygen‐deficient environment was established by the continuous nitrogen supply. Transfection was then conducted after 24 hours using the Lipofectamine 2000 kit (11668‐019; Invitrogen Inc) in a 5% CO_2_, 37°C incubator for 18 hours. The medium was replaced with fresh complete medium, and the cells were collected after 48 hours of transfection. The transfection efficiency was measured by RT‐qPCR.

### 2.20 Cell transfection efficiency determination

Cardiomyocytes were isolated from the mice and cultured in vitro. The cells were seeded in a 6‐well plate with M199 medium containing 10% FBS and cultured in an incubator with 5% CO_2_ at 37°C. When the cell confluence reached approximately 80%, the cells were transfected with pCDNA3.1‐enhanced green fluorescent protein (eGFP; 3 mg/well) with pCDNA3.1 as a control in accordance with the instructions of X‐tremeGENE HP DNA Transfection Reagent (Hoffmann‐La Roche Ltd.). After 48 hours of transfection, the positive rate of eGFP‐labelled cardiomyocytes was determined by a flow cytometer. The results (Figure S1) showed that the primary cardiomyocytes from the mice were well‐transfected, and the positive rate was 68.3%.

### 2.21 Dual‐luciferase reporter gene assay

Human embryo kidney 293T cell (HEK293T; AT‐1592; American Type Culture Collection) cells were seeded in a 24‐well plate and cultured for 24 hours. Arepsicially synthesized segments of NFKBIA‐wild‐type (WT) 3'untranslated region (UTR) and NFKBIA‐mutant (MUT) 3'UTR were separately introduced to construct the NFKBIA dual‐luciferase reporter gene vectors (pmiRRB‐NFKBIA‐3 'UTR). The idenepsied WT and MUT vectors were cotransfected into HEK‐293T cells with mouse ATP2B1‐AS1, siRNA‐mouse ATP2B1‐AS1 or the NC plasmid. After 48 hours of transfection, the luciferase activity was determined using Dual‐Luciferase^®^ Reporter Assay System (E1910; Promega Corporation).

### 2.22 3‐(4,5‐dimethylthiazol‐2‐yl)‐2,5‐diphenyltetrazolium bromide (MTT) assay

Cardiomyocytes were seeded into the 96‐well plates at a density of 1 × 10^4^ cells/well, with 8 parallel wells settled. The blank control was settled with culture medium only. Once reached 70% confluence, the cells in each well were cultured with 10 μL 5 mg/mL MTT solution (ST316; Beyotime) in an incubator at 37°C. After 4 hours, the cells in each well were treated with 100 μL dimethyl sulfoxide (DMSO; D5879; Sigma‐Aldrich) for 10 minutes. The OD value was measured at 490 nm using a microplate (MK3, Thermo). The cell viability was calculated as follows: cell viability = (OD value of the experimental well − OD value of the blank well)/OD value of the blank well.

### 2.23 cTNT and Ki67 double fluorescence staining

Cardiomyocytes were seeded in a 24‐well plate at 1 × 10^5^ cells/well, and a sterile coverslip was added to the bottom of each well. The cells were cultured in an incubator at 37°C for 12 hours and transfected with the mouse ATP2B1‐AS1 vector, NFKBIA vector, siRNA‐mouse ATP2B1‐AS1 and siRNA‐NFKBIA. Then, the cells were fixed by 10% paraformaldehyde for 30 minutes and permeabilized with sealing solution containing 0.1% Triton X‐100 (B1276; Westang) and 1% FBS for 1 hour. After that, the cells were incubated with rabbit antibody to Ki67 (ab15580; 1:200, Abcam) and mouse antibody to cTNT (ab8295, 1:200, Abcam) at 4°C overnight. Subsequently, the cells were incubated with diluted secondary fluorescent antibody (1:400) at 37°C for 30 minutes and stained with diluted DAPI (1:20) at 37°C for 30 minutes. The staining was visualized under the fluorescence microscope.

### 2.24 Flow cytometry

At 48 hours after transfection, the cells detached with 0.25% trypsin were harvested and centrifuged at 1000 g for 5 minutes. Subsequently, the cells were centrifuged again at 1000 g for 5 minutes and fixed with pre‐cooled 70% ethanol at 4°C overnight, and centrifuged at 1000 g for 5 minutes. Then, the cells were incubated with 10 µL RNase at 37°C for 5 minutes and stained with 1% propidium iodide (PI; Qian Chen Bioscience & Technologies Co., Ltd.) for 30 minutes in the dark. Finally, the cells were transferred to a flow cytometer (FACSCalibur, BD, FL, NJ) to assess the cell cycle by recording the red fluorescence at 488 nm.

At 48 hours after transfection, the cells were detached with ethylenediaminetetraacetic acid‐free trypsin. According to the manufacturer's instructions from the Annexin V‐fluorescein isothiocyanate (FITC)/PI cell apoptosis detection kit (CA1020; Solarbio), cell apoptosis was determined.

### 2.25 Enzyme‐linked immunosorbent assay (ELISA)

Cardiomyocytes were seeded in 96‐well plates. When the cells reached 70% confluence, the cell supernatant was harvested. According to the specifications of ELISA kits (ab137994, Abcam Inc), the concentration of interleukin 1β (IL‐1β), interleukin‐6 (IL‐6), interleukin‐10 (IL‐10) and tumour necrosis factor α (TNF‐α) in the supernatant was calculated through a linear regression equation of the standard curve.

### 2.26 Statistical analysis

All statistical analyses were performed with SPSS 21.0 software (IBM Corp.). The measurement data were expressed as the mean ± standard deviation. Comparisons between two groups were analysed using *t* test, whereas comparisons among multiple groups were performed using one‐way analysis of variance. The enumeration data were presented as a ratio or percentage, and the comparisons were analysed by a *chi*‐square test. *P* < .05 was considered as statistically significant.

## 3 RESULTS

### 3.1 LINC00936 is selected as study subject

**Figure 1:**
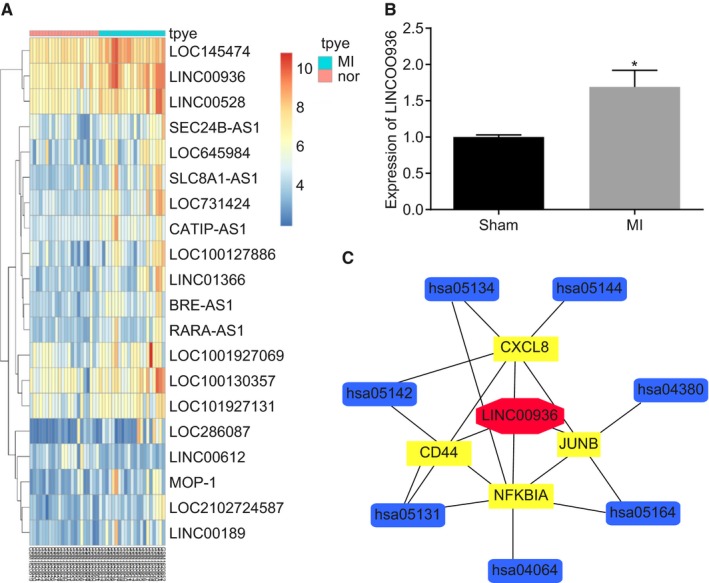
Involvement of LINC00936 and NFKBIA in MI based on a microarray analysis. A, the expression of LINC00936 in circulating endothelial cells isolated from AMI patients and healthy cohorts in the http://www.ncbi.nlm.nih.gov/geo/query/acc.cgi?acc=GSE66360 data set retrieved from GEO database (http://www.ncbi.nlm.nih.gov/geo/); B, the LINC00936 expression in the peripheral blood of healthy people and AMI in the http://www.ncbi.nlm.nih.gov/geo/query/acc.cgi?acc=GSE97320 data set retrieved from GEO database (http://www.ncbi.nlm.nih.gov/geo/); C, predicted targets of LINC00936; *P < .05 vs the sham group; LINC00936, long intergenic non‐protein‐coding RNA 936; MI, myocardial infarction; AMI, acute myocardial infarction; GEO, Gene Expression Omnibus

Based on the computer retrieval results from GEO database, we found that there were four MI‐related data sets (http://www.ncbi.nlm.nih.gov/geo/query/acc.cgi?acc=GSE66360, http://www.ncbi.nlm.nih.gov/geo/query/acc.cgi?acc=GSE48060, http://www.ncbi.nlm.nih.gov/geo/query/acc.cgi?acc=GSE65705 and http://www.ncbi.nlm.nih.gov/geo/query/acc.cgi?acc=GSE97320). The http://www.ncbi.nlm.nih.gov/geo/query/acc.cgi?acc=GSE48060 data set without differentially expressed lncRNAs and the http://www.ncbi.nlm.nih.gov/geo/query/acc.cgi?acc=GSE65705 data set with only two non‐MI control samples were excluded, while http://www.ncbi.nlm.nih.gov/geo/query/acc.cgi?acc=GSE66360 data set analysis revealed that LINC00936 showed increased expression in a largest fold in MI as compared to counterpart (Figure 1A). Based on the http://www.ncbi.nlm.nih.gov/geo/query/acc.cgi?acc=GSE97320 data analysis, the expression of LINC00936 was also remarkably higher in the patients with MI when compared to the healthy controls (Figure 1B). Therefore, LINC00936 was selected as the subject in the present study.

### 3.2 NFKBIA is regulated by LINC00936 and implicated in the NF‐κB signalling pathway

**Figure 2:**
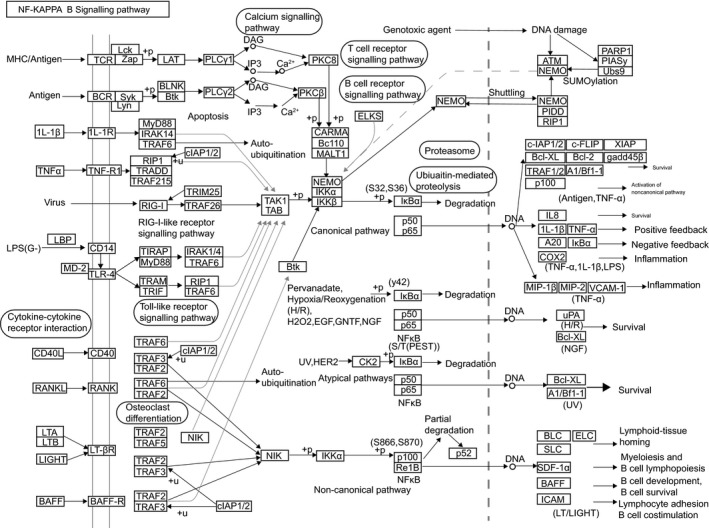
NFKBIA is involved in the NF‐κB signalling pathway. NFKBIA, NF‐kappa‐B inhibitor alpha

From the MEM website, four genes were obtained as putative targets of LINC00936. In addition, KEGG enrichment analyses in the WebGestalt website revealed that NFKBIA was implicated in the NF‐κB signalling pathway (Figure 1C). Among these 4 genes, NFKBIA was not only regulated by LINC00936, but also a central element in the NF‐κB signalling pathway. NFKBIA played protective roles in myocardial injury by relieving inflammation through the inhibition of the NF‐κB signalling pathway (Figure 2). In addition, the information retrieval of these 4 genes in MI revealed that the involvement of CXCL8, CD44 and JUNB in the functional mechanism of MI has been reported,^28^, ^29^, ^30^, ^31^, ^32^, ^33^ while few reports have investigated the role of NFKBIA in MI. Additionally, as NFKBIA was crucially involved in the NF‐κB signalling pathway, further investigations on NFKBIA are needed to acquire further insight into the functional mechanism of the NF‐κB signalling pathway in MI. Therefore, the above data demonstrated that NFKBIA was a target of LINC00936 with a key role implicated in the NF‐κB signalling pathway.

### 3.3 Mouse ATP2B1‐AS1 is evolutionarily conserved in mouse and human

**Figure 3:**
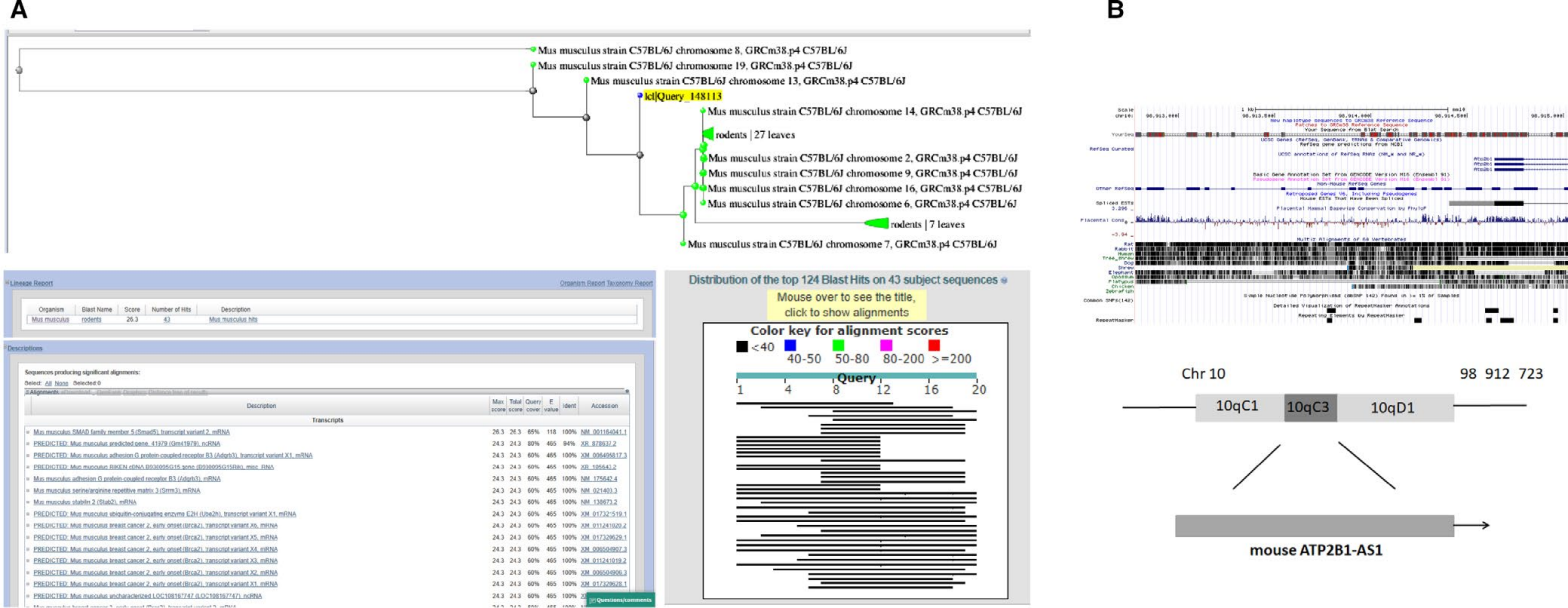
Mouse ATP2B1‐AS1 is evolutionarily conserved in mouse and humans. A, Homology of ATP2B1‐AS1 between human and mouse compared with BLASTN; B, ATP2B1‐AS1 localization in mouse genome by bioinformatics website; ATP2B1‐AS1, ATPase plasma membrane Ca^2+^ transporting 1 antisense RNA 1

The full‐length nucleotide sequence of mouse ATP2B1‐AS1 was retrieved from the Mouse Genome Database using BLASTN. The results showed that homologous sequences of this lncRNA were found in the mouse and human genomes (Figure 3A). At the same time, RNA‐seq data of SRA database and the ArrayExpress database were employed. The bioinformatics software TopHat and Cufflinks were used to compare the data and reconstruct the transcriptome, and the coding potential calculation software to analyse the transcripts. A transcript of mouse ATP2B1‐AS1 was screened. The location prediction of mouse ATP2B1‐AS1 in the mouse genome (Version: GRCm38/mm10) by USSC database (http://genome.ucsc.edu/) revealed that it was mainly located in the mouse chromosome 10 (Figure 3B). These results indicated that mouse ATP2B1‐AS1 was evolutionarily conserved in both mouse and humans.

### 3.4 The MI mouse model is successfully established

**Figure 4:**
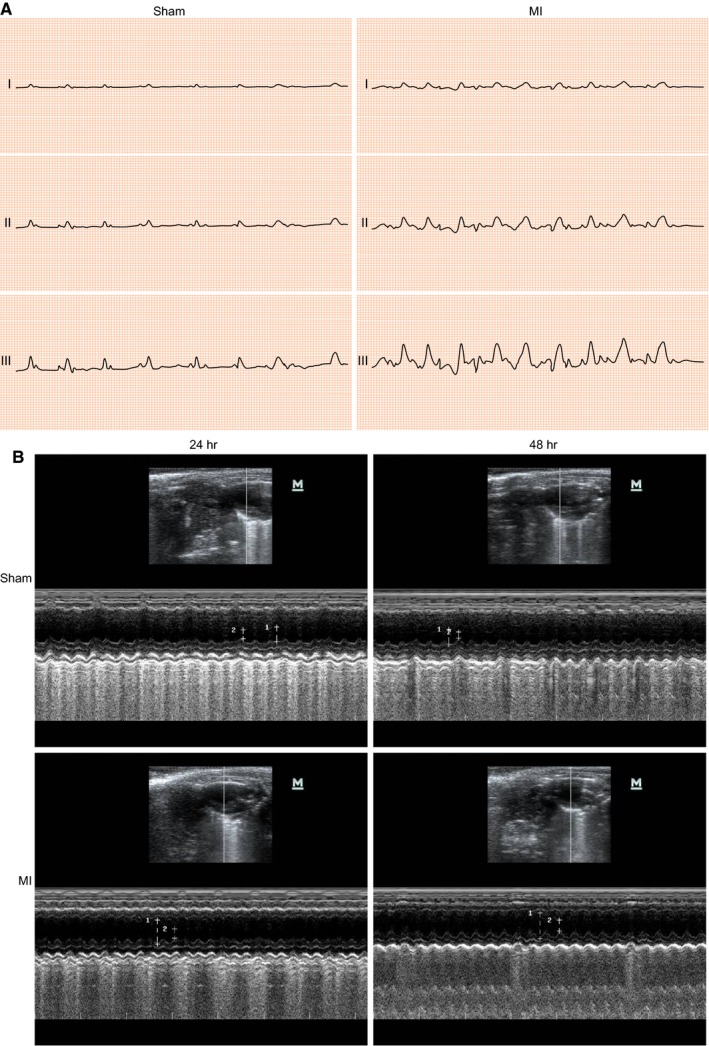
The successful establishment of MI mice is confirmed by ECG and UCG test. A, ECG of MI mice and sham‐operated mice after surgery (the excitation and repolarization width is 100 ms; sensitivity is 10 mm/mV; and chart speed is 50 mm/s); B, representative M‐mode images for both sham‐operated and MI mice by UCG. ECG, electrocardiograph; MI, myocardial infarction; UCG, ultrasonic cardiogram

Electrocardiogram was used to examine the ST‐segment elevation in the mouse model with MI. The results showed significant ST‐segment elevation and significant increases in amplitude of T‐wave and lead II of ECG (Figure 4A). Twenty‐four and 48 hours after surgery, the UCG test was performed to evaluate cardiac function to ensure the induction of MI (Figure 4B). Meanwhile, LVEDD, LVFS and LVESD were assessed before and after surgery and ejection fraction (EF), fractional shortening (FS) was evaluated. The results showed that EF, FS and left ventricular systolic diameter were remarkably reduced 48 hours after surgery. In the sham group, no statistically significant differences between before and after surgery were observed in LVEDD, LVESD, or LVFS. However, post‐operative LVEDD and LVESD were increased in the MI group, compared with the pre‐operative LVEDD and LVESD. Additionally, the ventricular anterior wall became thinner, and LVFS was reduced after operation (Table 2). In the MI group, the successful rate of model establishment within 2 weeks was 63.33% (19/30), among which 15 mice were chosen for further experiments. There were no deaths among the 15 mice in the sham group. Thus, these results indicated the successful establishment of the MI mouse models.

**Table 2:** UCG detection of FS, EF, LVEDD, LVESD and LVFS before and after surgery

Indexes	Sham		*P*	MI		*P*
	Pre‐operative	Post‐operative		Pre‐operative	Post‐operative	
FS	28.65 ± 2.79	30.22 ± 3.13	>.05	28.78 ± 3.03	14.96 ± 1.51	<.001
EF	56.17 ± 5.87	60.28 ± 6.34	>.05	56.49 ± 1.23	32.15 ± 2.09	<.001
LVEDD (mm)	3.69 ± 0.37	3.74 ± 0.33	>.05	4.01 ± 0.45	5.11 ± 0.45	<.001
LVESD (mm)	1.75 ± 0.27	1.71 ± 0.30	>.05	1.79 ± 0.19	3.74 ± 0.37	<.001
LVFS (%)	51.96 ± 9.24	54.26 ± 7.16	>.05	54.69 ± 7.86	26.16 ± 10.75	<.001

Note

LVFS = (LVEDD − LVESD)/LVEDD × 100%.

Abbreviations: EF, ejection fraction; FS, fractional shortening; LVEDD, left ventricular end‐diastolic diameter (mm); LVESD, left ventricular end‐systolic diameter (mm); LVFS, left ventricular fractional shortening (%); UCG, ultrasonic cardiogram.

### 3.5 LINC00936 is highly expressed, NFKBIA is poorly expressed and the NF‐κB signalling pathway is activated in the successful MI mouse model

Initially, morphology changes of cardiomyocytes were observed in the mice from both sham and MI groups. The results (Figure S2A) showed that 2 weeks after MI, there was no infarction in the mice in the sham group, while a greyish‐white area was obviously observed in the myocardial tissues from the mice in the MI group, and an infarct area of 25.54 ± 1.15% was determined. In addition, the cardiomyocytes from the sham group were neat and close in the arrangement, with an oval‐shaped nucleus in the cell and regularly arranged myocardial fibres without inflammatory cell infiltration and myocardial necrosis. On the other hand, the MI group presented a disorderly arranged and swollen cells, karyopyknosis, myocardial fibre ablation, and severe inflammatory cell infiltration with massive necrotic cardiomyocytes (Figure S2B). Under an optical microscope, the blue cell nucleus was visible in the myocardial tissues from the mice in the sham group, while many positive cells with green‐stained cell nuclei were observed in the MI tissues, indicating that many apoptotic cardiomyocytes were presented in the MI tissues (Figure S2C). These data displayed that the MI mouse model was successfully established.

The immunohistochemistry, RT‐qPCR and Western blot analyses were performed to assess the NFKBIA‐positive rate, mouse ATP2B1‐AS1 expression, and mRNA and protein levels of NFKBIA, NF‐κBp50, NF‐κBp65 and IKKβ. The immunohistochemical staining results (Figure S2D) showed that the NFKBIA protein was mainly expressed in the cytoplasm and stained yellow‐brownish. The MI mice exhibited a remarkable reduction of NFKBIA‐positive cells and significant decrease in average OD values in contrast to the sham‐operated ones (*P* < .05). The results of the RT‐qPCR and Western blot analyses suggested that when compared with the sham group, the MI group had a prominently up‐regulated mouse ATP2B1‐AS1 expression and elevated mRNA and protein levels of NF‐κBp50, NF‐κBp65 and IKKβ but reduced mRNA and protein levels of NFKBIA (all *P* < .05) (Figure S2E‐F). These results together demonstrated that mouse ATP2B1‐AS1 was highly expressed, while NFKBIA was poorly expressed and the NF‐κB signalling pathway was aberrantly activated in MI mice.

### 3.6 Mouse ATP2B1‐AS1 is highly expressed in myocardial fibroblasts and cardiomyocytes in MI mice

RT‐qPCR was employed to analyse the mouse ATP2B1‐AS1 expression in cardiomyocytes, myocardial fibroblasts, endothelial cells and PBMCs from heart tissues of MI mice. The results Figure S3) demonstrated that mouse ATP2B1‐AS1 expression was higher in the myocardial fibroblasts and cardiomyocytes, and partial expression was detected in the endothelial cells and PBMCs.

### 3.7 Cardiomyocytes are successfully isolated from neonatal mice

The morphology of cardiomyocytes was observed and idenepsied under a microscope. The results (Figure S4) showed that the cardiomyocytes were initially oval‐shaped, began to extend once the cells attached, forming a polygonal or spindle shape with a good refractive index. To confirm that the isolated cells were mouse cardiomyocytes, we used a neonatal mouse and observed the cTNT positive rate of cells isolated and cultured in vitro under a fluorescence microscope. The results revealed that the isolated cells emitted strong green fluorescence signals, suggesting that these isolated cells were cardiomyocytes.

### 3.8 siRNA and overexpression plasmids are successfully delivered

The transfection efficiency of the siRNAs and overexpression plasmids in the mice was determined by RT‐qPCR. The interference effect of siRNA‐mouse ATP2B1‐AS1‐1 was the best among the three types of siRNA‐mouse ATP2B1‐AS1 (Figure S5A), while siRNA‐NFKBIA‐3 was the best among the three types of siRNA‐NFKBI (Figure S5B). When compared with the NC group, an increased ATP2B1‐AS1 expression was observed in the mouse ATP2B1‐AS1 vector group but a down‐regulated ATP2B1‐AS1 expression observed in the siRNA‐mouse ATP2B1‐AS1 group (Figure S5C), while the mRNA level of NFKBIA was obviously increased in the NFKBIA vector group but remarkably decreased in the siRNA‐NFKBIA group (*P* < .05, Figure S5D), suggestive of successful transfection.

### 3.9 Mouse ATP2B1‐AS1 targets NFKBIA

The website lncRNATargets predicted binding sites between mouse ATP2B1‐AS1 and NFKBIA 3'UTR (Figure S6A), and showed that mouse ATP2B1‐AS1 could target NFKBIA. As shown in Figure S6B, when compared with the NC group, the luciferase activity of the NFKBIA‐WT 3'UTR was decreased in the mouse ATP2B1‐AS1 overexpression group but increased in the siRNA‐mouse ATP2B1‐AS1 group (*P* < .05), while no change was observed in the luciferase activity of the NFKBIA‐MUT 3'UTR (*P* > .05). These findings suggested NFKBIA as a target of mouse ATP2B1‐AS1.

### 3.10 Mouse ATP2B1‐AS1 restrains the expression of NFKBIA and activates the NF‐κB signalling pathway

The expression of ATP2B1‐AS1 and the mRNA and protein levels of NFKBIA and NF‐κB signalling pathway‐related proteins in the transfected cardiomyocytes were determined by RT‐qPCR (Figure S7A) and Western blot analyses (Figure S7B‐C). When compared with the blank and NC groups, the mouse ATP2B1‐AS1 vector group and mouse ATP2B1‐AS1 vector + NFKBIA vector group had significantly increased the expression of mouse ATP2B1‐AS1 (*P* < .05), while the siRNA‐mouse ATP2B1‐AS1 group and siRNA‐mouse ATP2B1‐AS1 + siRNA‐NFKBIA group had decreased expression of mouse ATP2B1‐AS1 (*P* < .05). Moreover, the NFKBIA vector group and siRNA‐mouse ATP2B1‐AS1 group had increased mRNA and protein levels of NFKBIA (*P* < .05), but the mouse ATP2B1‐AS1 vector group and siRNA‐NFKBIA group had decreased the mRNA and protein level of NFKBIA vs the blank and NC groups (*P* < .05). Consequently, either ATP2B1‐AS1 overexpression or siRNA‐mediated silencing of NFKBIA resulted in increased mRNA and protein levels of NF‐κBp50, NF‐κBp65 and IKKβ, but these parameters were decreased by either NFKBIA overexpression or siRNA‐mediated silencing of ATP2B1‐AS1 (*P* < .05). In contrast to the mouse ATP2B1‐AS1 vector group, the mouse ATP2B1‐AS1 vector + NFKBIA vector group demonstrated an increased in mRNA and protein levels of NFKBIA but reductions in NF‐κBp50, NF‐κBp65 and IKKβ (*P* < .05). Compared with the siRNA‐mouse ATP2B1‐AS1 group, the siRNA‐mouse ATP2B1‐AS1 + siRNA‐NFKBIA group revealed decreased mRNA and protein levels of NFKBIA but elevated levels of NF‐κBp50, NF‐κBp65 and IKKβ (*P* < .05). As a result, mouse ATP2B1‐AS1 inhibited the expression of NFKBIA and activated the NF‐κB signalling pathway. Furthermore, the overexpression of NFKBIA could reverse the up‐regulation of NF‐κB signalling pathway‐related proteins induced by the high expression of mouse ATP2B1‐AS1.

### 3.11 Mouse ATP2B1‐AS1 suppresses, while NFKBIA promotes cardiomyocyte proliferation

The viability of the transfected cardiomyocytes was measured by MTT. As shown in Figure S8A, when compared with the blank and NC groups, the cell viability was significantly decreased in the mouse ATP2B1‐AS1 vector and siRNA‐NFKBIA groups (*P* < .05), but significantly increased in the siRNA‐mouse ATP2B1‐AS1 group and NFKBIA vector group (*P* < .05). When compared with the mouse ATP2B1‐AS1 vector group, the mouse ATP2B1‐AS1 vector + NFKBIA vector group revealed an enhanced cell viability (*P* < .05). When compared with the siRNA‐mouse ATP2B1‐AS1 group, the siRNA‐mouse ATP2B1‐AS1 + siRNA‐NFKBIA group displayed a significant reduction in cell viability (*P* < .05). These results indicated that mouse ATP2B1‐AS1 inhibited the growth of cardiomyocytes, while NFKBIA promoted the growth of cardiomyocytes, and the overexpression of NFKBIA could reverse the inhibition of cardiomyocyte activity triggered by the overexpression of mouse ATP2B1‐AS1.

As shown in Figure S8B, the cardiomyocytes were marked by cTNT, the viable cells were marked by ki67, and the cell nucleus was marked by DAPI. In contrast to the blank and NC groups, the mouse ATP2B1‐AS1 vector and siRNA‐NFKBIA groups showed a significant reduction in cell viability, while the siRNA‐mouse ATP2B1‐AS1 and NFKBIA vector groups showed a marked increase in cell viability. When compared with the mouse ATP2B1‐AS1 vector group, the mouse ATP2B1‐AS1 vector + NFKBIA vector group displayed a prominently elevation in cell viability. When compared with the siRNA‐mouse ATP2B1‐AS1 group, the cell viability in the siRNA‐mouse ATP2B1‐AS1 + siRNA‐NFKBIA group was significantly decreased. The results of cTNT and ki67 double fluorescence staining further confirmed the results obtained by MTT assay. Taken together, the growth of cardiomyocytes was inhibited by ATP2B1‐AS1‐mediated down‐regulation of NFKBIA.

### 3.12 Mouse ATP2B1‐AS1 enhances, while NFKBIA inhibits cardiomyocyte apoptosis

The cell cycle distribution (Figure S9A) and apoptosis (Figure S9B) of the transfected cardiomyocytes were detected by flow cytometry. When compared with the blank and NC groups, the proportion of cells in the G1 phase and the apoptosis rate were progressively increased, but the proportion of cells in the S phase was decreased in the mouse ATP2B1‐AS1 vector and siRNA‐NFKBIA groups (*P* < .05). By contrast, the proportion of cells in the G1 phase and the apoptosis rate were decreased, and the proportion of cells in the S phase was increased in the siRNA‐mouse ATP2B1‐AS1 and NFKBIA vector groups (*P* < .05). When compared with the mouse ATP2B1‐AS1 vector group, the mouse ATP2B1‐AS1 vector + NFKBIA vector group showed reduced proportion of cells in the G1 phase and apoptosis rate but an increased proportion of cells in the S phase (*P* < .05). When compared with the siRNA‐mouse ATP2B1‐AS1 group, the siRNA‐mouse ATP2B1‐AS1 + siRNA‐NFKBIA group showed increased proportion of cells in the G1 phase and apoptosis rate but a decreased proportion of cells in the S phase (*P* < .05). These results indicated that mouse ATP2B1‐AS1 promoted cell apoptosis, but NFKBIA inhibited cell apoptosis, and the overexpression of NFKBIA could reverse apoptosis which induced by enhancement of mouse ATP2B1‐AS1 in cardiomyocytes.

### 3.13 Mouse ATP2B1‐AS1 promotes, while NFKBIA inhibits the release of inflammatory cytokines

The levels of inflammatory cytokines in the transfected cardiomyocytes were determined by ELISA. When compared with the blank and NC groups, the mouse ATP2B1‐AS1 vector and siRNA‐NFKBIA groups showed increased levels of IL‐1β, IL‐6 and TNF‐α, and a decreased level of IL‐10 (*P* < .05), but the siRNA‐mouse ATP2B1‐AS1 and NFKBIA vector groups displayed reduced levels of IL‐1β, IL‐6 and TNF‐α, and increased level of IL‐10 (*P* < .05). When compared with the mouse ATP2B1‐AS1 vector group, the mouse ATP2B1‐AS1 vector + NFKBIA vector group revealed decreased levels of IL‐1β, IL‐6 and TNF‐α, but an increased level of IL‐10 (*P* < .05). When compared with the siRNA‐mouse ATP2B1‐AS1 group, the siRNA‐mouse ATP2B1‐AS1 + siRNA‐NFKBIA group demonstrated elevations in the levels of IL‐1β, IL‐6 and TNF‐α, but a decreased level of IL‐10 (*P* < .05; Table 3). These results indicated that mouse ATP2B1‐AS1 promoted the release of inflammatory cytokines, but NFKBIA suppressed the release of inflammatory cytokines, and the overexpression of NFKBIA could reverse the up‐regulation of inflammatory cytokines caused by the overexpression of mouse ATP2B1‐AS1.

**Table 3:** Expression of inflammatory cytokines in transfected myocardial cells in different groups after transfection (pg/mL)

Group	IL‐1β	IL‐6	IL‐10	TNF‐α
Blank group	82.46 ± 8.42	126.78 ± 13.04	67.84 ± 7.15	302.14 ± 17.2
NC group	82.78 ± 8.25	125.89 ± 12.77	68.02 ± 7.22	299.54 ± 17.42
Mouse ATP2B1‐AS1 vector group	130.25 ± 12.43*	176.94 ± 16.12*	32.14 ± 5.40*	428.56 ± 16.88*
NFKBIA vector group	50.12 ± 5.24*, &	75.66 ± 8.03*, &	124.76 ± 11.55*, &	80.15 ± 8.32*, &
siRNA‐mouse ATP2B1‐AS1 group	51.36 ± 6.77*, &	80.42 ± 9.54*, &	118.65 ± 10.12*, &	94.67 ± 10.98*, &
siRNA‐NFKBIA group	142.74 ± 5.22*, #	183.65 ± 20.15*, #	35.12 ± 4.65*, #	415.88 ± 20.15*, #
Mouse ATP2B1‐AS1 vector + NFKBIA vector group	88.96 ± 8.05^&^, ^#^	120.72 ± 10.63^&^, ^#^	70.22 ± 7.12^&^, ^#^	286.32 ± 15.44^&^, ^#^
siRNA‐mouse ATP2B1‐AS1 + siRNA‐NFKBIA group	85.42 ± 8.78^&^, ^#^	132.95 ± 14.18^&^, ^#^	68.37 ± 6.72^&^, ^#^	314.75 ± 18.97^&^, ^#^

Abbreviations: ATP2B1‐AS1, ATPase plasma membrane Ca^2+^ transporting 1; IL‐1β, interleukin 1β; IL‐6, interleukin‐6; NC, negative control; NFKBIA, NF‐kappa‐B inhibitor alpha; siRNA, small interfering RNA; TNF‐α, tumour necrosis factor α.

* *P* < .05 vs the blank and NC groups.

^&^ *P* < .05 vs the mouse ATP2B1‐AS1 vector group.

^#^ *P* < .05 vs the siRNA‐mouse ATP2B1‐AS1 group.

## 4 DISCUSSION

Although treatment and prevention of MI have achieved great progress in the past few years, patients with MI still face a modest risk of heart failure due to the failed compensation of apoptotic cardiomyocytes.^8^ The whole‐genome transcriptome analysis has showed that many cardiac‐specific lncRNAs involve in many unique functions and regulations related to myocardial remodelling, myocardial regeneration and heart function.^34^ This study was conducted to investigate the role of mouse ATP2B1‐AS1 in MI based on its related bioinformatics information. The results conclusively suggested that the overexpression of NFKBIA induced by silencing of mouse ATP2B1‐AS1 may protect against MI by inhibiting the NF‐κB signalling pathway in mice.

Initially, poor expression of NFKBIA but high expression of mouse ATP2B1‐AS1 was witnessed in MI. It has indicated that lncRNAs not only exert a great influence on various pathobiologies of human diseases, but also regulate gene expression at the epigenetic, transcriptional and post‐transcriptional levels.^35^ However, only a few lncRNAs have been reported to be involved in the cardiovascular system.^36^ LncRNA metastasis‐associated lung adenocarcinoma transcript 1 (MALAT1) has been found to be up‐regulated in MI.^9^ Differentially expressed lnRNAs are determined in MI and co‐expressed with differentially expressed mRNAs, and their interactions are implicated in pathogenesis mechanism of MI.^37^ Silencing of ATP2B1‐AS1 can suppress renal interstitial fibrosis and oxidative stress in chronic renal failure.^10^ In this study, mouse ATP2B1‐AS1 silencing was suggested to inhibit the cardiomyocyte apoptosis and inflammation, suggesting a potential of ATP2B1‐AS1 in protection against MI. In this study, based on a target prediction programme and the dual‐luciferase activity determination, we found that mouse ATP2B1‐AS1 targeted NFKBIA. In addition, a reduction in MI susceptibility has been shown to be related to the −94 ins/del ATTG NFKB1 gene variant.^38^ We speculated that ATP2B1‐AS1 might target NFKBIA to play a regulatory role in MI.

In addition, we found that mouse ATP2B1‐AS1 silencing or NFKBIA overexpression resulted in reduced levels of NF‐κBp50, NF‐κBp65 and IKKβ, suggestive of blockade of the NF‐κB signalling pathway. The activation of NF‐κB was triggered by IκBα degradation *via* phosphorylation by the IκB kinase complex.^39^ NF‐κB activation has been found in other different heart diseases, including cardiac hypertrophy, diabetic cardiomyopathy, myocardial infarction, ischaemia‐reperfusion injury and heart failure.^40^ The signalling pathways that mediate NF‐κB activation could be divided into canonical pathways (mainly p50‐RelA) and non‐canonical (mainly p52‐RelB) pathways.^41^ The canonical pathway is related to a great variety of stimuli, including both endogenous and exogenous ligands and physical and chemical stress plethora.^42^ The activation of IKK could result in the phosphorylation of IκBα at two N‐terminal serines to trigger its proteasomal degradation and ubiquitination, thus causing the nuclear translocation of NF‐κB complexes, including p50/RelA and p50/c‐Rel dimers.^43^ The non‐canonical NF‐κB pathway mainly relies on p52/RelB NF‐κB complex activation by inducing the processing of p100, which is a molecule that acts as both an inhibitor of RelB and precursor of p52.^44^ The disruption of the NF‐κB signalling pathway contributes to reduced post‐infarct myocardial remodelling.^45^ In addition to impairing the function of NF‐κB by inhibiting its binding to DNA, NFKBIA also suppresses NF‐κB by masking the nuclear localization signals of NF‐κB protein and allows it to remain inactive in the cytoplasm.^11^ Our findings mainly suggested that ATP2B1‐AS1 targeted NFKBIA and consequently activated the NF‐κB signalling pathway.

Of crucial importance, this study demonstrated that NFKBIA overexpression restrained the cardiomyocyte apoptosis and the production of inflammatory cytokines, while enhancing cardiomyocyte proliferation through the blockade of the NF‐κB signalling pathway. NF‐κB most likely acts as a key mediator of immune and inflammatory responses and is involved in stress responses as well as the regulation of cell proliferation and apoptosis.^46^ Another study has revealed that the reduced NF‐κB activity is resulted from the overexpression of IkB could attenuate post‐infarct remodelling and improve the cardiac function.^47^ Additionally, NFKBIA has been demonstrated to be associated with altered NF‐κB activities, thus mediating carcinogenesis in nasopharyngeal carcinoma.^48^ A published article has demonstrated that mouse ATP2B1‐AS1 overexpression significantly inhibits multiple myeloma cell growth and has a negative impact on genes involved in the mTOR signalling pathway.^49^ Regarding our results, NFKBIA could reverse both the inhibition of cardiomyocyte viability and the up‐regulation of the inflammatory cytokines expression which was triggered by mouse ATP2B1‐AS1. Hence, ATP2B1‐AS1‐mediated repression of NFKBIA activated the NF‐κB signalling pathway, thus enhancing cardiomyocyte apoptosis.

In conclusion, this study provides evidence that the knocking down mouse ATP2B1‐AS1 may protect against MI‐induced cell apoptosis and inflammation by inhibiting the NF‐κB pathway through the up‐regulation of NFKBIA expression (Figure S10). However, due to the limited time, space and study subjects, this study was not highly comprehensive for translation into clinical application. Extensive studies should be conducted to further investigate the multiple target sites, as well as the clinical target therapy related to mouse ATP2B1‐AS1 in the future.

## CONFLICT OF INTERESTS

None.

## AUTHOR CONTRIBUTION

Kai‐You Song, Xian‐Zhao Zhang, Feng Li and Qing‐Rong Ji designed the study. Kai‐You Song collated the data, designed and developed the database, carried out data analyses and produced the initial draft of the manuscript. Xian‐Zhao Zhang, Qing‐Rong Ji and Feng Li contributed to drafting the manuscript. All authors have read and approved the final submitted manuscript.

## Supporting information

Fig S1

Fig S2

Fig S3

Fig S4

Fig S5

Fig S6

Fig S7

Fig S8

Fig S9

Fig S10

## Data Availability

The data that support the findings of this study are available from the corresponding author upon reasonable request.
